# Prognostic Reliability of a New Classification System for Blount’s Disease

**DOI:** 10.7759/cureus.8353

**Published:** 2020-05-29

**Authors:** Achraf H Jardaly, Michael Conklin, Shane F Strom, Kevin C Wall, Shawn Gilbert

**Affiliations:** 1 Orthopaedic Surgery, University of Alabama at Birmingham, Birmingham, USA

**Keywords:** blount's disease, tibia vara, recurrence, classification system, prognosis, langenskiold classification, lamont classification

## Abstract

Objective

We conducted this study to evaluate the reproducibility of a new classification system for Blount's disease and assess its correlation with established radiological measures used to evaluate the severity of this disorder.

Materials and Methods

This is a retrospective review of children with Blount’s disease that were younger than 10 years of age. Recurrence was defined as the need for a second corrective surgery. Radiographs immediately pre-surgery and at final follow-up were used to measure mechanical axis (MA), tibial metaphyseal-diaphyseal angle (TMDA), epiphyseal-metaphyseal angle (EMA), lateral distal femoral angle (LDFA), and medial proximal tibial angle (MPTA). Patients were stratified according to the new classification (Type A, B, or C).

Results

Sixty-five limbs from 16 males and 24 females met our inclusion criteria. The average follow-up was 4.2 years. Twelve patients (with 22 Type-A extremities) underwent bracing with a success rate of 54%. Thirty-four patients (53 extremities) underwent surgical correction. The recurrence rate was 35.8%. Group C had a recurrence rate of 62%, higher than that of Group B (33%), and Group A (23%) (P = 0.026). In addition, irrespective of reoperation, patients in Group C had the least change in the MA (62%, P = 0.046) and the most severe values of MPTA and TMDA initially and after the operation (P < 0.05).

Conclusion

The new classification system for Blount’s disease holds validity for predicting recurrence. The severity of the grades is correlated with the TMDA, MPTA, and varus reversibility. This can aid physicians and families in making an informed decision and setting treatment goals.

## Introduction

Blount’s disease is a pediatric condition in which patients develop a pathologic form of genu varum [1]. It is postulated that this disease is closely related to obesity due to biomechanical overload inhibiting the posteromedial tibial physis [2-3]. As the prevalence of childhood obesity is increasing, more children are predicted to suffer from Blount’s, and a better understanding of the disorder is needed to treat patients optimally [4].

Given the degree of similarity in age and presentation of patients with Blount’s and those with physiologic bowing, significant emphasis has been placed on accurately diagnosing the disease [1, 5]. In particular, radiographs have been shown to reliably predict disease development and progression, and certain radiological measures, such as the tibial metaphyseal-diaphyseal angle (TMDA) and the epiphyseal-metaphyseal angle (EMA), have been associated with disease progression [6]. The Langenskiöld classification, a progressive six-stage scale that relies on the radiographic appearance of the tibia metaphysis and epiphysis, has been widely used to group patients depending on disease severity [7]. This classification system is of prognostic importance, where the more advanced stages correlate with more extensive surgery and a higher risk of recurrence after the presumed corrective surgery - tibial osteotomy with or without epiphysiolysis [1]. However, many patients with only Langenskiöld Stage II or III disease also suffer from recurrence. To address this issue, LaMont et al. proposed a new classification, which assigned patients to one of three types based on the radiographic presence and extent of the medial metaphyseal/epiphyseal defect and sloping in children with tibia vara [8]. They found that Type C patients, those with increased tibial vertical sloping, experienced worse outcomes following corrective surgery and had a higher recurrence rate. This new classification system helped to more accurately delineate the high-risk, LaMont Type C patients, from the lower risk Types A and B patients than the Langenskiöld classification was able to do, in which the aforementioned high rates of recurrence, even at its lower stages, made it less strong of a prognostic tool [8].

Prognosis is of high importance for both patients and physicians as it helps anticipate outcomes and provide individualized care. Having an accurate classification system that is of prognostic ability is invaluable. We conducted this retrospective study to evaluate the reproducibility of the LaMont classification system and assess its correlation with established radiological measures used to evaluate the severity of Blount’s disease.

## Materials and methods

The electronic medical records and radiographs of patients with Blount’s disease were reviewed after Institutional Review Board approval. Inclusion criteria were idiopathic Blount’s disease in children younger than 10 years of age. Patients were excluded if they had other causes of varus angulation (e.g., posttraumatic, metabolic bone disease), incomplete records, or inadequate follow-up.

Information gathered included patient age, sex, weight, laterality, and type of intervention (bracing and/or surgery). Brace success was defined as avoiding the need for surgical correction. In patients undergoing operative management, recurrence was defined as the need for a subsequent surgery for deformity correction. Radiographs were reviewed at the earliest available (initial) timepoint, immediately pre- and post-intervention, and at the last available (final) follow-up. In some patients, the initial presentation was also the pre-intervention timepoint. Similarly, some patients had only a single postoperative visit and so the final time point was the same as the immediate post-intervention timepoint.

**Figure 1:**
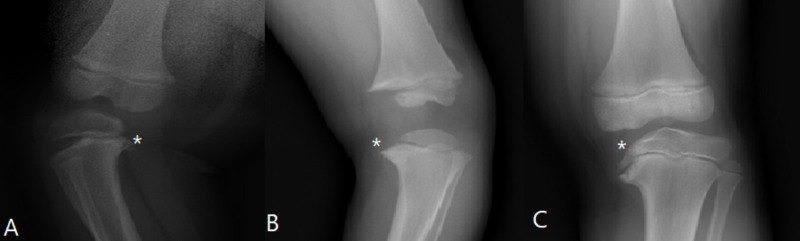
X-rays demonstrating an example of Types A, B, and C according to the LaMont et al. classification for Blount’s disease LaMont et al. [8]

Radiographic measures included the mechanical axis (MA), tibial metaphyseal-diaphyseal angle (TMDA), epiphyseal-metaphyseal angle (EMA), lateral distal femoral angle (LDFA), and medial proximal tibial angle (MPTA). The percentage change of the MA was calculated as the (initial MA-final MA)/initial MA. The radiographs of each time point were additionally classified into Types A, B, or C according to the classification of Lamont et al. In Type A, there is a metaphyseal defect that is partially lucent. Type B shows increased lucency of the epiphysis and metaphysis such that the metaphyseal beak is downsloping with an upturned medial border. In Type C, there is downward sloping of both the epiphysis and metaphysis such that the metaphyseal down-sloping is not followed by an upturned medial border. An example of each of the types is presented in Figure 1.

Cases were assigned to the LaMont classification system by a single, experienced pediatric orthopaedist, and the radiographic measurements were performed by two observers. 

Chi-square, two-sample t-test, and analysis of variance (ANOVA) were used when appropriate to determine statistical significance, with a threshold for α set at 0.05.

## Results

Sixty-seven patients younger than 10 years of age with Blount’s disease were identified from our institution over a 19-year period. Twenty-seven were excluded due to incomplete medical records or radiographs that were unavailable from at least one time point. Our study included 65 limbs from 16 males and 24 females. Patients were followed up for an average of 4.2 years (range: 11 months - 11.5 years). The intraclass correlation of coefficient was greater than 0.9 for each of the five angles measured, indicating an almost perfect agreement, and the average difference of measurements was 2˚. 

Twelve patients (22 extremities) underwent bracing. All were 30 months or younger at the date of brace initiation and were categorized as LaMont Type A. Bracing was successful in 12 extremities, yielding a success rate of 54%. No differences in the age or radiographic parameters were observed between patients who had success or failure with bracing (Table 1). Bilaterality was also not different between both groups (P = 0.31). 

**Table 1:** Characteristics of Patients Undergoing Brace Treatment EMA: epiphyseal-metaphyseal angle; LDFA: lateral distal femoral angle; MA: mechanical axis; MPTA: medial proximal tibial angle; Pre: immediate pre-surgery; TMDA: tibial metaphyseal-diaphyseal angle

	Successful (N = 12)		Failed (N = 10)		P-value
	Mean	Range	Mean	Range	
Age (months)	24.6	21 - 30	23.6	17 - 30	0.71
Pre-MA, degrees	31	20 - 45	29	9 - 53	0.72
Pre-TMDA, degrees	16	10 - 20	13	5 - 19	0.16
Pre-EMA, degrees	23	17 - 32	24	17 - 30	0.63
Pre-LDFA, degrees	106	71 - 116	104	88 - 118	0.69
Pre-MPTA, degrees	79	71 - 88	76	64 - 87	0.23

Thirty-four patients (53 extremities) underwent surgical correction for Blount’s disease. This includes the 10 extremities that failed bracing. One child had a normal weight, one was overweight, and the remaining were obese with a weight greater than the 97th percentile. The recurrence rate was 35.8%. Among the 19 patients requiring a second surgery, five cases were due to the persistence of the varus deformity postoperatively rather than true recurrence. These five cases occurred with guided growth, whereas all patients treated with an osteotomy (with acute or gradual correction) initially corrected after surgery. The pre-treatment MPTA was 9˚ larger in patients who did not develop recurrence (P = 0.010), and the pre-treatment TMDA was 5˚smaller (P = 0.03) (Table 2). The other radiographic parameters and age were not statistically significantly different between both the recurrence and no recurrence groups.

**Table 2:** Characteristics of Patients Undergoing Surgical Correction of Blount’s Disease Values in bold are statistically significant (P < 0.05). EMA: epiphyseal-metaphyseal angle; LDFA: lateral distal femoral angle; MA: mechanical axis; MPTA: medial proximal tibial angle; Pre: immediate pre-surgery; TMDA: tibial metaphyseal-diaphyseal angle

	No Recurrence (N = 33)		Recurrence (N = 20)		P-value
	Mean	Range	Mean	Range	
Age (years)	6.0	1.8 - 9.7	6.1	3.2 - 9.9	0.94
Pre-MA, degrees	23	3 - 49	28	12 - 56	0.22
Pre-TMDA, degrees	13	3 - 25	18	4 - 37	0.030
Pre-EMA, degrees	23	13 - 40	26	14 - 43	0.14
Pre-LDFA, degrees	95	77 - 112	92	85 - 102	0.20
Pre-MPTA, degrees	74	54 - 95	65	41 - 79	0.010

**Figure 2:**
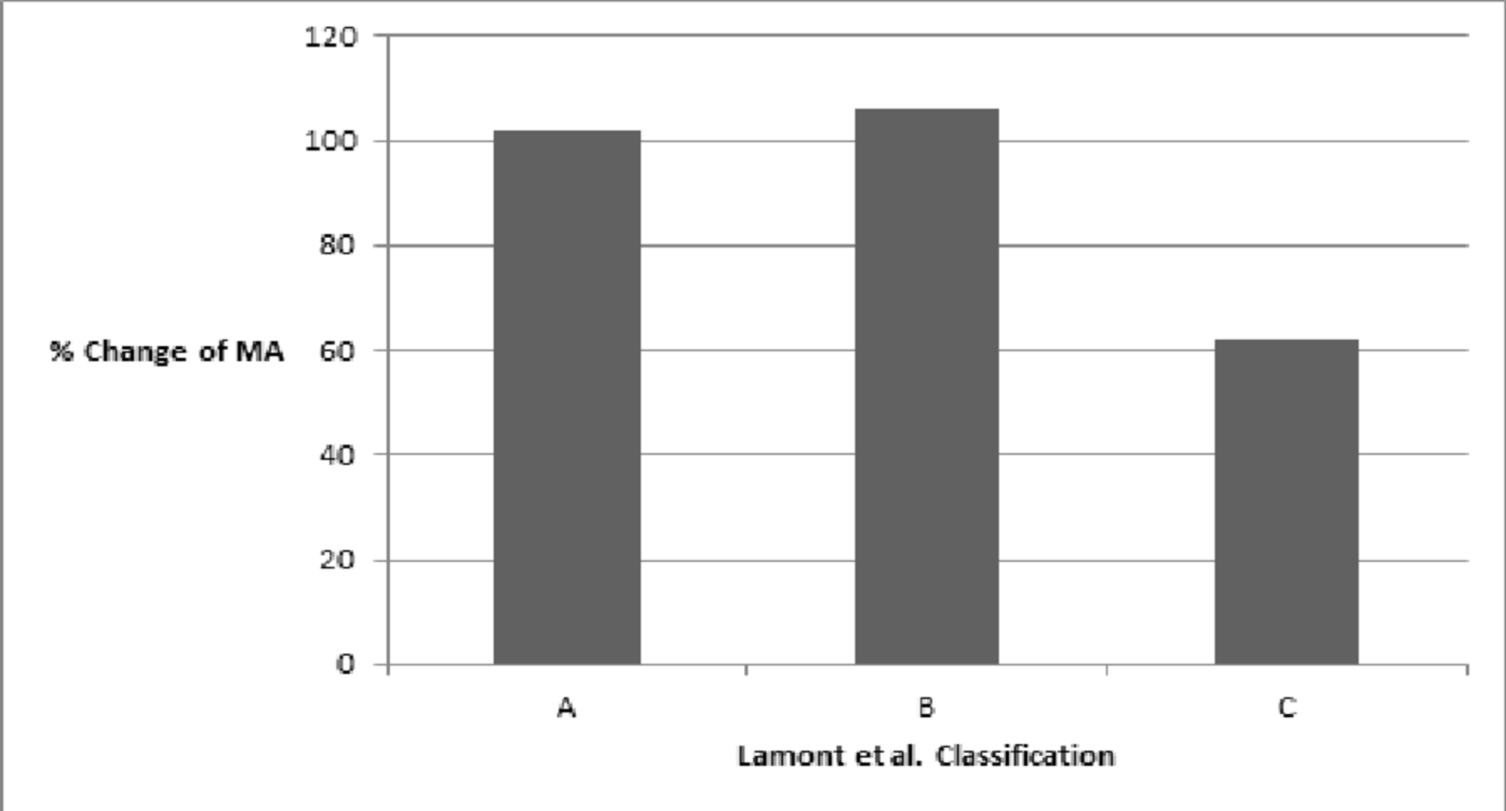
The change of the mechanical axis (MA) based on the new classification

**Figure 3:**
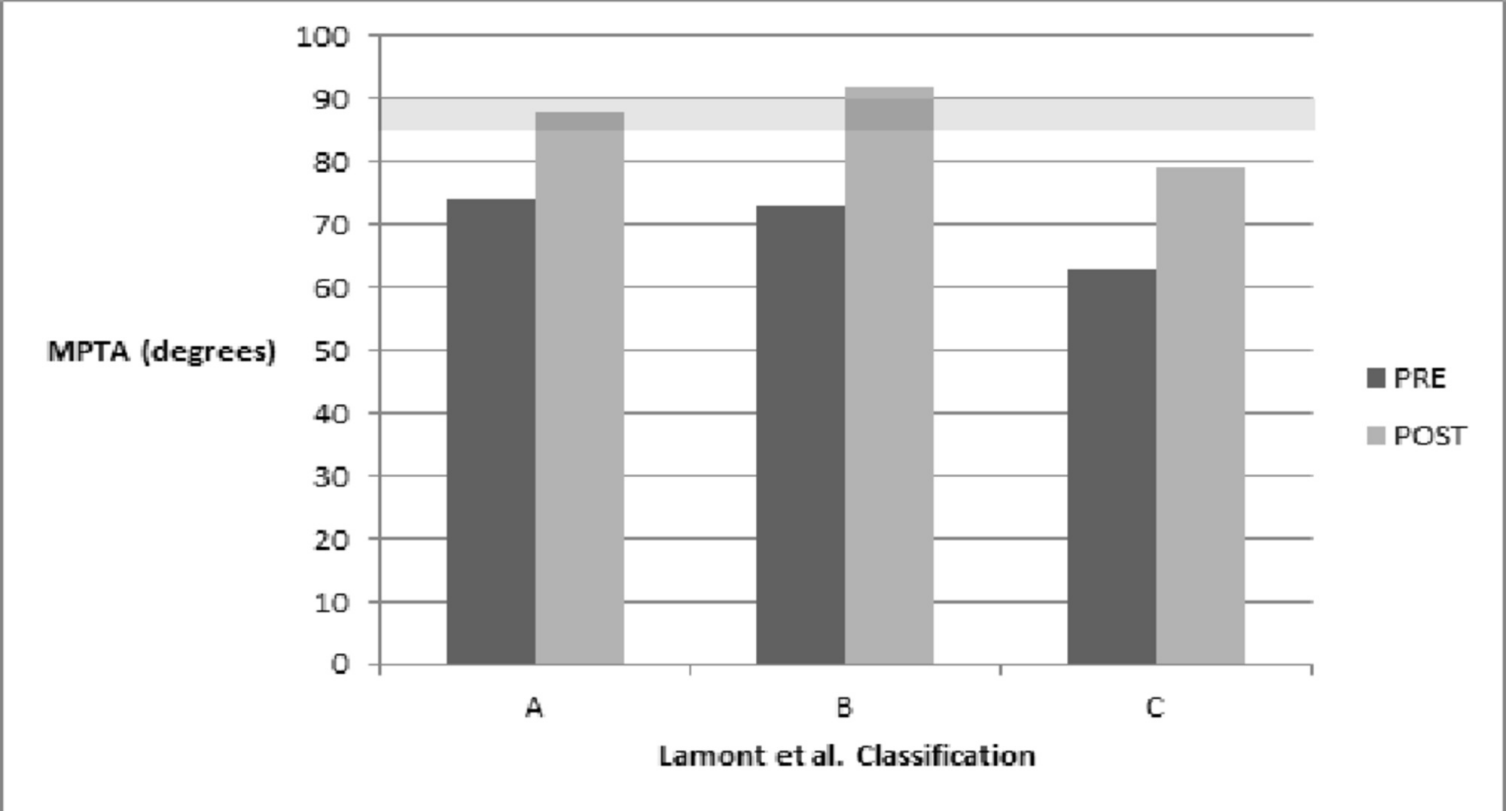
Initial and final medial proximal tibial angle (MPTA) The shaded area represents the normal range of the MPTA (85˚-90˚) Pre: immediate pre-surgery; Post: final follow-up

**Figure 4:**
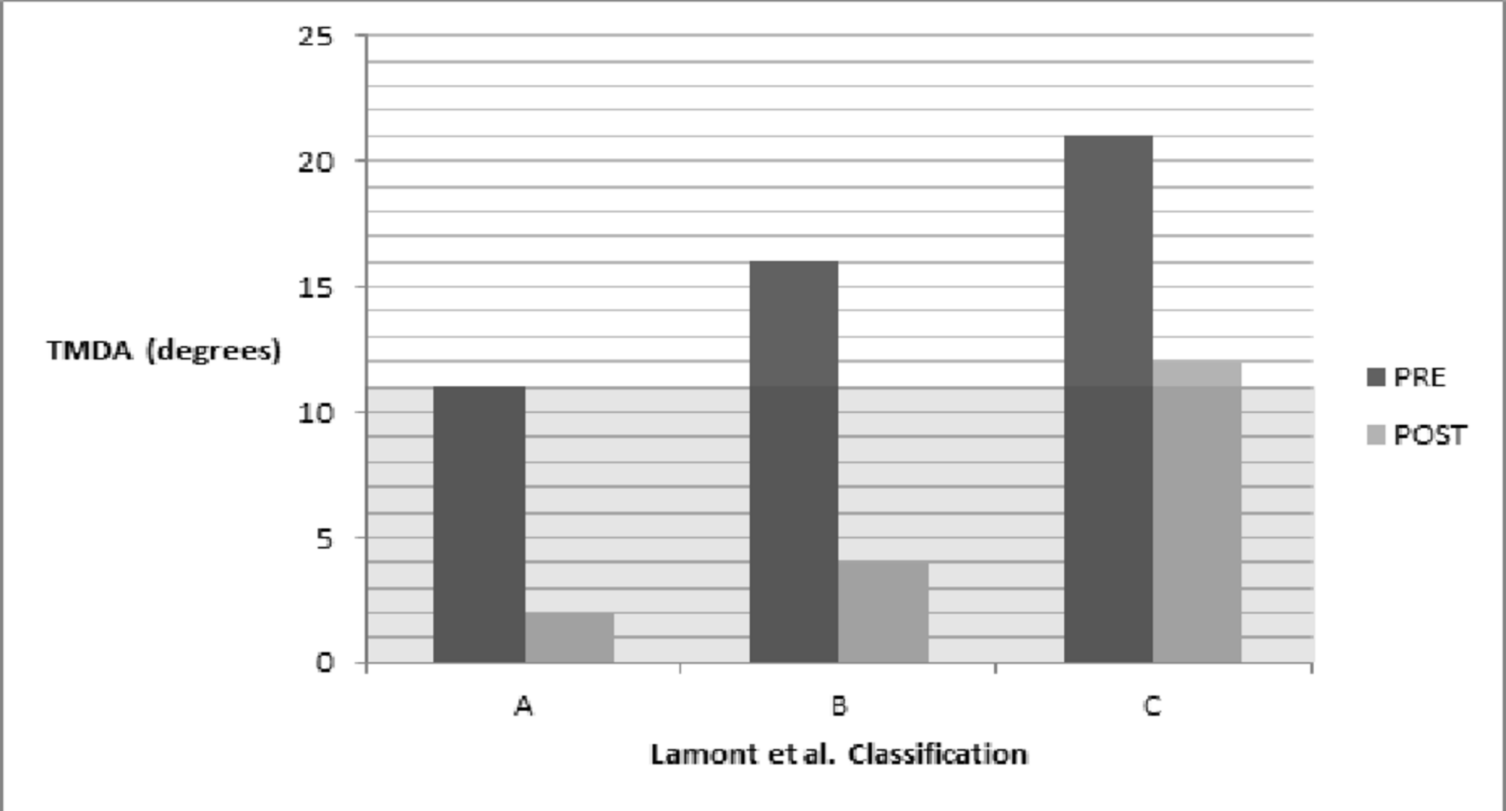
Initial and final tibial metaphyseal diaphyseal angle (TMDA) The shaded area represents the normal value for the TMDA (≤ 11˚). Pre: Immediate pre-surgery; Post: final follow-up

Based on the LaMont classification, 31 extremities belonged to Type A, six to Type B, and 16 to Type C. Type C had a recurrence rate of 62%, higher than that of Type B (33%), and Type A (23%). This difference was statistically significant (P = 0.026). Four cases had a final varus ≥ 5˚ but did not undergo a second procedure. Two of the cases belonged to Type A and two to Type C. In addition, irrespective of reoperation, patients in Group C had the least percentage change in the MA (62%, P = 0.046), the lowest values of the initial and final MPTA (63˚, P = 0.01 and 79˚, P = 0.0067, respectively), and the largest values of the initial and final TMDA (21˚, P = 0.0001 and 12˚, P = 0.0008) (Figures 2-4).

Prior studies demonstrated that recurrence rates vary according to age (cutoff of four years) and type of surgery [9-10]. Stratifying our patients by age, those younger than four years had a recurrence rate of 32%, and those four years of age and older had a rate of 41% (P = 0.49). Recurrence occurred in 31% of guided growth procedures, 36% of tibial osteotomies with acute correction, and 46% of osteotomies with gradual correction (P = 0.76). Guided growth was the most common procedure performed. Recurrence after this procedure was 29% in Type A, 33% in Type B, and 60% in Type C (P = 0.41).

## Discussion

Children with Blount’s disease can benefit from conservative and operative measures. Bracing is a viable option for children three years and younger [10]. Previous studies demonstrate that bracing improves alignment and avoids the need for corrective surgery in 50% to 90% of children [11-13]. In the current study, 54% of the bracing cohort experienced success with treatment. Bilaterality, which has been previously reported as a risk for brace failure, was not a predictor of failure in the present series. The initial MA, EMA, and TMDA were not different between patients experiencing success and failure with bracing. All patients braced in the current study were in Group A of the LaMont classification. Therefore, we cannot draw conclusions about the usefulness of this classification in predicting brace success. Additional research is needed to assess for clinical and radiological factors that might predict responsiveness to bracing.

Since body mass index (BMI) can predict the development of Blount’s disease, it might also be a factor affecting brace success [13-14]. Unfortunately, the BMI was not available for our brace cohort. The natural history of infantile Blount’s remains unclear [15]. One study demonstrated high rates of spontaneous resolution of the disease, questioning the use of bracing; however, it is clear that once the disease has reached a certain stage, resolution without treatment is not expected [1]. Bracing is a safe procedure, and our patients did not experience complications from brace treatment. Though we employed daytime bracing for most children, when discomfort or compliance is an issue, nighttime bracing can also be used and has similar reported success rates [16].

Surgical correction is required when patients fail bracing or present at an older age or with a more severe deformity [1, 15, 17]. Despite advances in the surgical treatment of Blount’s disease, recurrence remains an issue, with reported rates as high as 88% [18-20]. A higher stage of the Langenskiöld classification is a commonly reported risk factor for recurrence [1]. More recently, the medial metaphysis has gained attention as a predictor of recurrence. Specifically, a larger medial metaphyseal slope has been shown to be associated with a higher chance of recurrence [21]. LaMont et al. proposed an alternative to the Langenskiöld classification to achieve greater prognostic accuracy [8]. The LaMont system classifies patients into Types A, B, and C based on the radiographic appearance of the medial metaphysis and epiphysis. Their study demonstrated that 71.7% of Type C patients had a recurrence, significantly greater than Types A (22.5%) and B (20.7%). We aimed to assess the reproducibility of these results. Indeed, patients belonging to Type C had a statistically significant higher recurrence rate than Types A and B in the current study with a reoperation rate of 62%. Furthermore, irrespective of recurrence, Types A and B managed to achieve a greater percentage of varus correction as demonstrated by a MA change of approximately 100%, indicating complete reversibility of the angular deformity in the earlier stages of the disease, while Type C achieved only a 62% change in the MA. Type C patients also presented with more severe deformity as measured by the initial MPTA (63˚, P = 0.01) and TMDA (21˚, P = 0.0001). Despite improvement with surgery, Type C patients remained the farthest from the normal ranges for the MPTA and TMDA, even after a second operation.

There are limitations associated with this study. Bracing compliance was not monitored, so our evaluation of bracing effectiveness is based on intent to treat. In our study, recurrence was defined by the need for repeat surgery in patients whose primary treatment was surgical correction. As such, patients who may have met treatment failure criteria but never received their appropriate subsequent treatment were not captured as failures. Our rationale for this study design is that it is the same as that used in the study by LaMont et al. and reproducing their methods yields the greatest degree of reliability between studies. Nonetheless, if we expand recurrence to include patients with a final varus ≥ 5˚, four cases will be added to the recurrence group, yielding an overall recurrence rate of 45.3%. The LaMont classification would still hold validity for predicting recurrence, with 66.7% of Type C cases experiencing recurrence as opposed to 33% of Type B and 30.3% of Type A (P = 0.022). A final limitation of this study is our moderate sample size and occasional cases of limited available data. A larger and more comprehensive sample may have clarified whether some trends are representative of true differences, for example, in regard to age and type of procedure.

## Conclusions

In conclusion, the new classification system for Blount’s disease is reproducible. It is a valid measure for predicting recurrence, and the severity of the grades is correlated with the TMDA and MPTA, which reinforces its validity. Additional studies are needed to assess its inter-observer reliability, but this classification can be useful in research and clinical settings.
